# DCLTV: An Improved Dual-Condition Diffusion Model for Laser-Visible Image Translation

**DOI:** 10.3390/s25030697

**Published:** 2025-01-24

**Authors:** Xiaoyu Zhang, Laixian Zhang, Huichao Guo, Haijing Zheng, Houpeng Sun, Yingchun Li, Rong Li, Chenglong Luan, Xiaoyun Tong

**Affiliations:** 1 Graduate School, Space Engineering University, Beijing 101416, China; 2 Key Laboratory of Intelligent Space TTC&O Ministry of Education, Space Engineering University, Beijing 101416, China; 3 National Key Laboratory of Space Target Awareness, Space Engineering University, Beijing 101416, China

**Keywords:** image translation, diffusion model, laser images, Brownian bridge

## Abstract

Laser active imaging systems can remedy the shortcomings of visible light imaging systems in difficult imaging circumstances, thereby attaining clear images. However, laser images exhibit significant modal discrepancy in contrast to the visible image, impeding human perception and computer processing. Consequently, it is necessary to translate laser images to visible images across modalities. Existing cross-modal image translation algorithms are plagued with issues, including difficult training and color bleeding. In recent studies, diffusion models have demonstrated superior image generation and translation abilities and been shown to be capable of generating high-quality images. To achieve more accurate laser-visible image translation, we designed an improved diffusion model, called DCLTV, which limits the randomness of diffusion models by means of dual-condition control. We incorporated the Brownian bridge strategy to serve as the first condition control and employed interpolation-based conditional injection to function as the second condition control. We also established a dataset comprising 665 pairs of laser-visible images to compensate for the data deficiency in the field of laser-visible image translation. Compared to five representative baseline models, namely Pix2pix, BigColor, CT2, ColorFormer, and DDColor, the proposed DCLTV achieved the best performance in terms of both qualitative and quantitative comparisons, realizing at least a 15.89% reduction in FID and at least a 22.02% reduction in LPIPS. We further validated the effectiveness of the dual conditions in DCLTV through ablation experiments, achieving the best results with an FID of 154.74 and an LPIPS of 0.379.

## 1. Introduction

With progress in science and technology, as well as the development of urbanization, smart communities have become an important direction in modern urban construction. Video monitoring systems play a key role in improving community safety and emergency response efficiency. Currently, mainstream monitoring equipment primarily utilizes visible light imaging systems for image acquisition, which is a passive imaging approach that exploits the target’s radiation or the reflection of other light sources to capture an image. As a result, this approach is highly susceptible to variations in light intensity, weather, and imaging distance. Consequently, the images captured by visible light imaging systems in the case of low illumination, rainy and foggy conditions, or long imaging distances tend to exhibit significant blurriness. In more serious cases, it may become nearly impossible to obtain images. Laser active imaging systems can compensate for these shortcomings by actively illuminating the field of view with a narrow-spectrum laser, thereby enabling clear imaging even under difficult imaging circumstances. The combination of visible light imaging systems and laser active imaging systems can realize all-day and all-weather image acquisition, which has extensive prospects for application in the security and surveillance domain. However, laser images are essentially grayscale images, and are plagued by drawbacks such as low contrast, the absence of color information, and indistinct edge features between targets and backgrounds. As a consequence, laser imaging demonstrates significant modal discrepancy compared to the visible image, which hinders the human interpretability of laser images. Existing image processing algorithms, such as image classification, image recognition, and object detection, are predominantly designed for visible images. They cannot be directly applied to processing laser images because of the modal discrepancy, which restricts the application of relevant advanced computer algorithms. Therefore, it is of great significance to propose a method capable of translating laser images into visible images.

The task of translating a laser image into a visible light image can be conceptualized as transferring the image from a source domain to a target domain, which falls within the domain of image translation [1,2,3,4,5,6,7,8,9,10]. More specifically, it is similar to image colorization in cross-modal image translation. Currently, the cross-modal translation of visible images primarily focuses on grayscale, infrared, and SAR images [11,12,13,14,15,16,17,18,19]. In contrast, research efforts dedicated to laser image translation remain scarce. Traditional cross-modal image translation methods can be primarily categorized into human-guided image translation [20,21] and reference-based image translation [22,23], both of which require substantial human effort and computational resources, while also exhibiting poor robustness.

With the development of deep learning and artificial intelligence-generated content (AIGC) technology, generative models have emerged as the mainstream methods in the field of image translation. The Generative Adversarial Network (GAN) [24] is the most classical generative model. It trains a generator to generate pseudo-images that closely align with the distribution of real images and trains a discriminator to determine whether the images are generated or authentic. Through an iterative process of adversarial learning and alternating optimization, the GAN employs a minimax game training methodology to enable the model to generate pseudo-images that closely approximate the distribution of real data. Pix2pix [11] proposes a general model for image-to-image translation by adding conditional guidance to the original GAN network. BigColor [13], based on BigGAN [25], enhances the colorization capability through the joint optimization of the encoder–generator module, as well as the discriminator, thereby enabling the model to learn a generative color prior. Qin et al. [26,27] further advanced the CycleGAN [28] network by optimizing its loss function and integrating spectral normalization, consequently achieving effective translation of laser face images. Nevertheless, GAN models generally suffer from unstable training and model collapse and cannot handle images with complex structures and semantics. Transformer [29] was particularly successful after its adoption in the field of natural language processing (NLP). Since then, Vision Transformer (ViT) [30] has also shown quite impressive performance and has been widely used in the field of computer vision, including image translation. InstaFormer [31] introduces an instance-level content contrastive loss by simultaneously considering instance-level features and global features, which provides the Transformer model with the ability to learn interactions between object instances and the global image. Ji et al. [14] proposed a novel color Transformer, ColorFormer, based on hybrid attention mechanisms and color memory. It introduced a global–local multi-scale attention operation to capture long-range dependencies and employs a color memory module to adaptively incorporate color priors into feature queries. However, some Transformer models require the training of several independent subnets, leading to accumulated error, while others may cause color bleeding when tackling complex image contexts [16].

Recently, diffusion models have demonstrated excellent performance in the field of image generation because of their impressive image generation capabilities, which is the main force in promoting the development of AIGC technology. Renowned models such as DALLE [32], SD [33], and Imagen [34] are all based on the diffusion models. The concept of diffusion models was initially proposed in [35]. Subsequently, the Denoising Diffusion Probabilistic Model (DDPM) [36] made improvements and became the state-of-the-art model in many fields because of its excellent density estimation as well as sample quality. As a likelihood-based model, diffusion models do not suffer from mode collapse and training instability like GAN models and outperform Transformer-based models in terms of efficiency when dealing with high dimensional data [33]. Diffusion models are capable of emulating extremely complex image distributions, making them a highly promising approach in the field of computer vision for handling intricate visual data. The core theory of diffusion models is derived from non-equilibrium thermodynamics and Markov chains. Diffusion models acquire the ability to generate high-quality images by initially subjecting the target image to a process of gradual noising until it is ultimately transformed into pure Gaussian noise, from which the target image is then reconstructed. Although these models are capable of generating images with considerable diversity, an inherent and unavoidable degree of unpredictability and randomness is also present. In the context of specific tasks of image translation, such as image super-resolution [37,38], inpainting [39,40], and image colorization [41,42], there is a tendency to retain the generative ability of diffusion models while simultaneously limiting the randomness, so as to generate more deterministic images that are better suited to the requirements of these particular tasks.

With the aim of fulfilling the task of laser-visible image translation, we explored the efficacy of diffusion models in this regard. In this paper, given the scarcity of research on laser image processing and the absence of publicly available laser-visible image data, we first constructed a laser-visible image dataset with the objective of remedying the deficiency of training data. Additionally, we propose an improved diffusion model utilizing two conditional control methods, which purposefully guides the noise prediction of the neural network by introducing extra conditional features to limit the randomness of diffusion models. Moreover, the self-attention mechanism was employed to further enhance the feature extraction capability of our model in high-dimensional space. Our major contributions and principal methodology are as follows:

1. We designed a laser-visible image acquisition system and constructed a dataset comprising one-to-one corresponding laser-visible images that can be used for both unsupervised and supervised methods.
2. An improved diffusion model, DCLTV, is proposed to tackle the laser-visible image translation challenge. The proposed model limits the random generation of diffusion models via dual conditions consisting of a Brownian bridge strategy and interpolation-based conditional injection. To the best of our knowledge, this is the first time that the diffusion model has been explored in the field of laser-visible image translation.
3. Compared to five representative baseline models, the proposed DCLTV achieves the best performance in both quantitative and qualitative experiments, which proves its effectiveness in laser-visible image translation.

## 2. Laser-Visible Image Dataset

In this section, we present the reasons and the theoretical approach for constructing the laser-visible image dataset. Additionally, we exhibit several image instances within the dataset.

### 2.1. Dataset Acquisition

In the field of AIGC, data are crucial for training highly effective deep learning neural network models capable of solving complex problems. Many studies have shown that the quality and scale of data directly affect the performance of neural network models. At present, publicly available image translation datasets mainly include visible light image datasets, SAR image datasets, and infrared image datasets. To the best of our knowledge, there is no available public dataset dedicated to laser-visible image translation, except for the laser-visible face dataset constructed by Qin et al. [26,27]. Therefore, to conduct a more comprehensive analysis, it was imperative to initially construct a laser-visible image dataset.

We obeyed the following rules when capturing images to ensure the standardization and validity of the dataset:

1. Diversity. The dataset ought to encompass as extensive a variety of scene and target categories as feasible so that the neural network models can better learn color and edge features in the real world.
2. Multiple angles. Given that an object may present radically different appearances when photographed from diverse angles, it is essential to capture images of the scenes and targets from multiple perspectives, which can make the dataset more comprehensive.
3. Matching. In order to satisfy the requirements of supervised and unsupervised methods and to accurately evaluate the models, laser images and visible images need to be acquired in a paired fashion, meaning that they must be captured in the same field of view and under the same environmental conditions.

The acquisition of visible images necessitates conducting the process under visible light illumination condition; thus, an outdoor experimental environment was chosen, where sunlight was available. Since the wavelengths of sunlight cover invisible (ultraviolet and infrared) and visible light bands, extraneous light outside the wavelengths of the laser source will exert a significant influence on the captured images during the execution of laser active imaging. Consequently, in order to obtain a suitable laser image, the impact of light from other bands should be minimized as much as possible.

After careful consideration, we decide to acquire data during the evening twilight period, a time when sunlight intensity is subdued yet outdoor ambient brightness remains adequate for capturing clear visible images. At the same time, the impact of sunlight on laser active imaging can be mitigated as much as possible. To ensure comprehensiveness, we endeavored to collect a wide variety of scenes and targets. The scenes primarily include street views, natural environments, and parking lots, while the targets mainly consisted of plants, trees, vehicles, buildings, bulletin boards, and landmarks. Moreover, we chose an imaging distance of between 50 m and 100 m.

**Figure 1:**
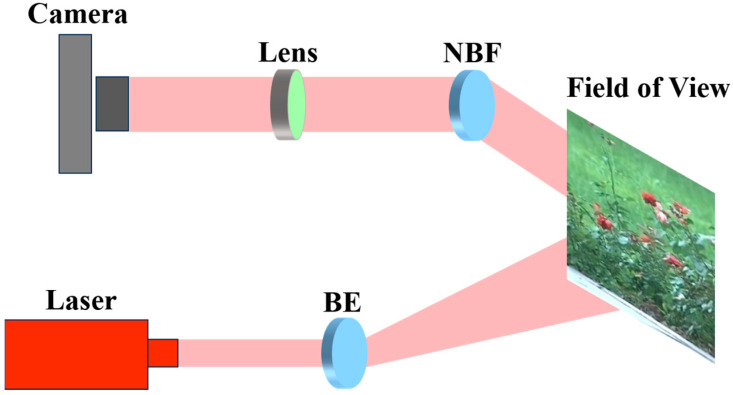
Flowchart of the laser-visible image acquisition system.

To construct the dataset in accordance with requirements, we designed a laser-visible image acquisition system primarily composed of five key components: a camera, an electric zoom lens, a laser, an electric beam expander, and a narrow band filter. The camera is a color CCD industrial camera capable of capturing both laser and visible images. The sensor size of the camera is 1/3″, with a frame rate of 30 FPS, and the captured image resolution is 1024 × 768. The electric zoom lens is a 400 mm lens with a focal length ranging from 10 mm to 400 mm, an aperture size of F2.8, and a field of view of 1/2″. The electric beam expander is utilized to increase the diameter and divergence angle of the laser beam, ensuring that the laser can cover the entire field of view. The laser is an 850 nm near-infrared laser used for active illumination of the field of view during the capture of laser images. The narrow band filter, with a central wavelength of 850 nm, is designed to reduce the influence of light from other wavelengths when performing laser active imaging. Figure 1 illustrates the overall flowchart of the acquisition system, where NBF and BE represent the narrow band filter and the electric beam expander, respectively.

### 2.2. Dataset Processing

The images captured through our image acquisition system possess a native resolution of 1024 × 768. Considering that prevalent image translation network models typically leverage datasets with image resolutions of 256 × 256 or 512 × 512 pixels, and in order to augment the scale of the dataset, we cropped the original images using image processing software. Following the principle of maintaining the quality of the image and ensuring the existence of relatively complete scenes and targets, we opt for a cropping resolution of 512 × 512 pixels for each image. To further expand the dataset, we employed a typical data augmentation method [43], mirror flipping, on the cropped images. Specifically, we conduct flipping along the horizontal axis and the vertical axis of each cropped image.

### 2.3. Instances of the Laser-Visible Image Dataset

**Figure 2:**
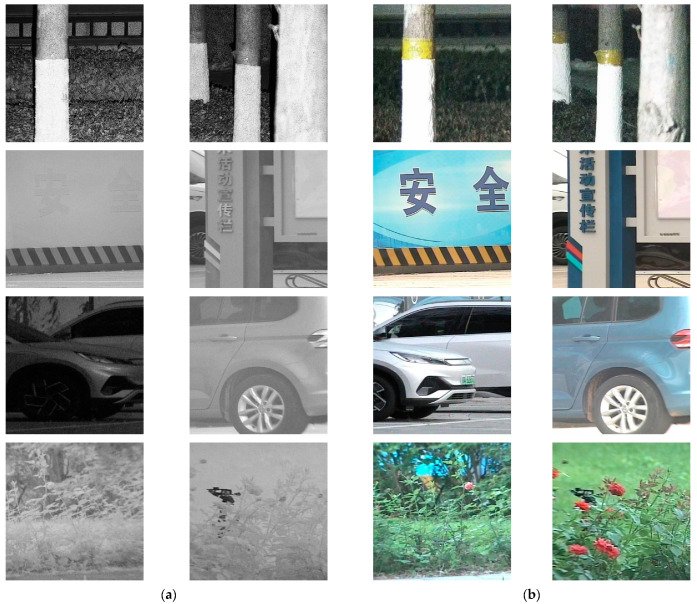
Examples taken from the laser-visible image dataset. (**a**) Laser images; (**b**) visible images.

We capture 100 laser images and 100 visible images, amounting to a total of 100 paired images. After the cropping described in Section 2.2, we obtain 250 pairs of laser-visible images. Among the 250 paired images, the three main scenes respectively accounted for roughly 30%, and there were approximately 50 images each of plants, trees, vehicles, buildings. Bulletin boards, landmarks, and other targets account for the remaining images. For the subsequent model training, the image dataset was divided into training and test sets in a 9:1 ratio, yielding 215 paired images for the training set and 35 paired images for the test set. To ensure the robustness of the network model, we only applied mirror flipping to the training set. Ultimately, our laser-visible image dataset comprised 630 paired training images and 35 paired test images. Figure 2 showcases some example images from the dataset.

## 3. Methods

In this section, we first introduce the overall framework and diffusion process of DCLTV, followed by a comprehensive elaboration covering the forward diffusion process, reverse diffusion process, and dual-condition control.

### 3.1. Overall Framework and Diffusion Process of DCLTV

**Figure 3:**
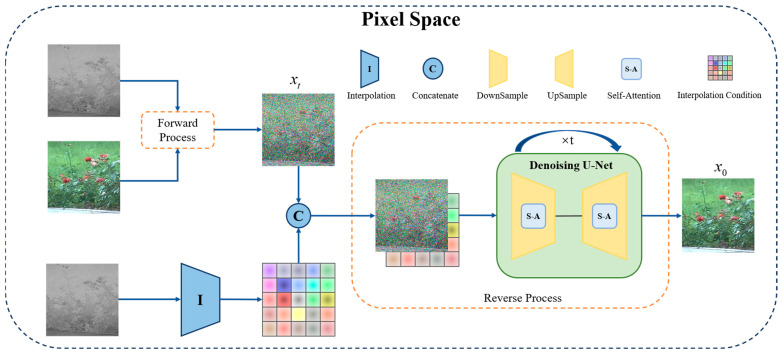
The model framework of DCLTV.

**Figure 4:**
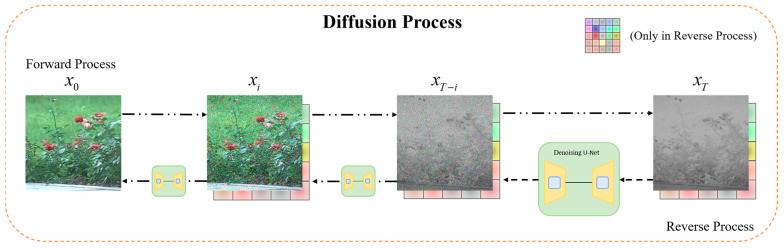
The diffusion process of DCLTV.

We designed an improved diffusion model based on dual-condition control to accomplish the translation of laser images to visible images, called DCLTV. Figure 3 illustrates the model’s main framework. Firstly, the input laser image and visible image were subjected to a forward process to gain a noised image, while the laser image was processed by interpolation to obtain interpolation conditional features. Then, the outputs from both processes were concatenated and fed into the network for training. DCLTV employs a U-Net network structure as a backbone, which is commonly used in diffusion models, incorporating a self-attention mechanism. Figure 4 shows the diffusion process flowchart of DCLTV, where the forward noise addition process begins on the left and the reverse denoising process begins on the right. It is noteworthy that the interpolation condition only participates in the reverse denoising process.

Given a pair of laser-visible images $\left(laser,visible\right)$, our goal was to learn the mapping relationship between the laser image domain and the visible image domain. In diffusion models, this typically involves a forward diffusion process and a reverse diffusion process. The forward diffusion process gradually adds random Gaussian noise to the target image until it is destroyed into a standard Gaussian distribution. The reverse diffusion process leverages a neural network to fit the conditional probability distribution $p_{\theta }\left(x_{t-1}|x_{t}\right)$, enabling the network to predict the random Gaussian noise added at each step and gradually denoise to restore the noised image into the target image, thereby learning the mapping relationship between the two image domains.

Traditional diffusion models generate images with remarkable diversity by sampling from pure Gaussian noise. However, certain specific tasks, such as laser-visible image translation, require more deterministic results. To achieve this, additional constraints need to be imposed on traditional diffusion models to reduce the randomness of the generated images, thus generating the desired colorful visible images. This is the source of inspiration for our proposed dual-condition control approach.

DCLTV also encompasses forward and reverse diffusion processes. However, we introduced the Brownian bridge principle during the diffusion process, transforming the noise addition endpoint (also the denoising starting point) from pure Gaussian noise to the target image, as shown in Figure 4 from left to right. This is our first conditional control. Additionally, during the reverse diffusion process, DCLTV integrates the noised image with the conditional features extracted via interpolation for guiding the generation towards the target domain, as shown in Figure 4 from right to left. This interpolation-based conditional injection is our second conditional control. A detailed exposition is provided the following sections.

### 3.2. Bridge Principle

The Brownian bridge is a common research object in probability theory, representing a continuous-time stochastic process that also conforms to the properties of the Markov chain, which is a special case of Brownian motion characterized by a fixed starting and ending state. For example, there is a set of random variables following a normal distribution $\left(x_{0},\ldots ,x_{T}\right)$ with time steps t ranging from 0 to T. Then, the Brownian bridge can be formulated as:

(1) $$p\left(x_{t}|x_{0},x_{T}\right)=N\left(\left(1-\frac{t}{T}\right)x_{0}+\frac{t}{T}x_{T},\frac{t(T-t)}{T}I\right)$$

It can be observed that, when t=0 and t=T, the entire process is fixed by $x_{0}$ and $x_{T}$, with the intermediate process forming a bridge between them.

### 3.3. Diffusion Process for DCLTV

#### 3.3.1. Forward Diffusion Process

Based on Markov chain theory, DDPM [36] defines a forward diffusion process starting from a source image data $x_{0}\sim q_{data}\left(x_{0}\right)$ and gradually adding noise until the source image data are transformed into standard Gaussian noise $x_{T}$. The entire process can be expressed as follows:

(2) $$q\left(x_{1},\ldots ,x_{T}|x_{0}\right)=\prod_{t=1}^{T}q\left(x_{t}|x_{t-1}\right)$$

(3) $$q\left(x_{t}|x_{t-1}\right)=N\left(x_{t};\sqrt{1-\beta_{t}}x_{t-1},\beta_{t}I\right)$$

where t is the time step, $\beta_{t}\in \left(0,1\right)$, and $q\left(x_{t}|x_{t-1}\right)$ denotes a normal distribution. During the reverse diffusion process, DDPM samples images from pure Gaussian noise by predicting the noise added at each step and then denoising it. Therefore, the images generated by DDPM have significant diversity and randomness. However, this is a disadvantage when learning the mapping between two deterministic domains. We aim to reduce the randomness in the sampling process to achieve a more deterministic end-to-end image generation diffusion model.

Inspired by Li et al. [44], we employ the Brownian bridge strategy to directly introduce conditional control during the noise addition and denoising processes for mitigating the randomness. Unlike DDPM, the forward diffusion process of DCLTV starts with the visible image and ends with the laser image. For consistency, we will denote the target image (visible image) as $x_{0}$ and the source image (laser image) as y in the following sections. The forward diffusion process of DCLTV can be formulated as follows:

(4) $$q_{BB}\left(x_{t}|x_{0},y\right)=N\left(x_{t};\left(1-m_{t}\right)x_{0}+m_{t}y,\delta_{t}I\right)$$

where $\delta_{t}$ represents the variance and $m_{t}=\frac{t}{T}$. Normally, according to Equation (1), the variance $\delta_{t}$ should be $\frac{t(T-t)}{T}$. However, when $t=\frac{T}{2}$, the variance at intermediate points will be extremely large as the total number of time steps T increases. This phenomenon contradicts the principle mentioned in [36,44,45] that, if $x_{0}$ satisfies a standard normal distribution, the variance at intermediate steps in the forward diffusion process should remain consistent. Furthermore, it may hinder model training. Therefore, we adopt a new variance design:

(5) $$\delta_{t}=1-\left({\left(1-m_{t}\right)}^{2}+m_{t}^{2}\right)=2\left(m_{t}-m_{t}^{2}\right)$$

When t=0, $m_{0}=0$, and the mean is the target image $x_{0}$. When t=T, $m_{T}=1$, and the mean is the source image y. According to Equation (5), the variance starts from 0 and gradually increases, reaching its maximum at the midpoint $t=\frac{T}{2}$, and then gradually decreases to 0. In the Brownian bridge process, we can adjust the diversity of the generated images by controlling the maximum variance at intermediate time steps. For this purpose, we can use a coefficient s to expand the variance $\delta_{t}$:

(6) $$\delta_{t}=2s\left(m_{t}-m_{t}^{2}\right)$$

According to Equation (4), we can obtain the marginal distribution for each time step t, but we also need to derive the forward transition probability from $x_{t-1}$ to $x_{t}$ during training and inference. Through recursive calculation, we can first obtain the following expression for $x_{t-1}$:

(7) $$x_{t-1}=\left(1-m_{t-1}\right)x_{0}+m_{t-1}y+\sqrt{\delta_{t-1}}\epsilon_{t-1}$$

where $\epsilon_{t}\sim N\left(0,I\right)$. According to Equation (4), the specific representation of the forward transition probability $q_{BB}\left(x_{t}|x_{t-1},y\right)$ is able to be obtained by substituting $x_{t-1}$ in Equation (7) for $x_{0}$ as follows:

(8) $$q_{BB}\left(x_{t}|x_{t-1},y\right)=N\left(x_{t};\frac{1-m_{t}}{1-m_{t-1}}x_{t-1}+\left(m_{t}-\frac{1-m_{t}}{1-m_{t-1}}m_{t-1}\right)y,\delta_{t|t-1}I\right)$$

where $\delta_{t|t-1}=\delta_{t}-\delta_{t-1}\frac{{\left(1-m_{t}\right)}^{2}}{{\left(1-m_{t-1}\right)}^{2}}$. Finally, the forward diffusion process of DCLTV from the target image $x_{0}$ to the source image y is established.

#### 3.3.2. Reverse Diffusion Process

Unlike most diffusion models, we forego the conventional approach of starting denoising from pure Gaussian noise. Instead, we turn the denoising starting point into the source image y through the Brownian bridge strategy, thereby directly incorporating conditions into the diffusion process. This approach can reduce the generative uncertainty of the diffusion models to better adapt to laser-visible image translation. According to the core idea of diffusion models, we can obtain the posterior probability required for the reverse process as follows:

(9) $$p_{\theta }\left(x_{t-1}|x_{t},y\right)=N\left(x_{t-1};\mu_{\theta }\left(x_{t},t\right),{\tilde{\delta }}_{t}I\right)$$

where $\mu_{\theta }\left(x_{t},t\right)$ is the mean of the noise distribution predicted by the neural network, and ${\tilde{\delta }}_{t}$ is the variance of the noise distribution at each time step. Instead of adopting a method that learns the variance in training as in ref. [46], we fix it as in traditional diffusion models. Although the variance is not learned, it is still important for the training effect of the model.

### 3.4. Interpolation-Based Conditional Injection

In our experiments, we found that merely relying on the Brownian bridge strategy to enhance the capability of diffusion model for laser-visible image translation was insufficient, leading to issues such as color bleeding and color deficiency in the generated images. The specific results and analysis are presented and discussed in Section 4.

To address the aforementioned issues, we contemplated whether it was feasible to impose further constraints on the diffusion model to generate more deterministic images. Unlike the diffusion model employing the Brownian bridge strategy, which directly injects conditions into the diffusion process to establish a connection between two domains, most diffusion models impose conditional restrictions by inputting additional conditions into the neural network model during the reverse diffusion process, thereby guiding the denoised images towards the target domain [33,47,48,49,50]. Drawing inspiration from this, we combined the strengths of both approaches and propose an improved diffusion model with dual conditions.

Inspired by [49], we considered feeding the encoded image of the laser image after interpolation into the neural network for conditional guidance. This improved method is commonly applied in super-resolution tasks, wherein the resolution of the source image is interpolated to align with the resolution of the target image, thereby achieving the desired level of detail and clarity. We believe that the interpolation approach in super-resolution essentially predicts the distribution of pixels around each pixel point of the source image, providing prior information for the neural network to better learn the connections among each patch of the image. On the basis of this, we encoded the laser image through a bicubic interpolation and then concatenated the encoded image with the noised image before inputting them into the neural network, providing supplementary conditional control to further mitigate the randomness of the diffusion model.

Taking $\overset{\wedge }{y}$ to denote the encoded image after interpolation, we obtained the reverse diffusion process as follows:

(10) $$p_{\theta }\left(x_{t-1}|x_{t},y\right)=N\left(x_{t-1};\mu_{\theta }\left(x_{t},\overset{\wedge }{y},t\right),{\tilde{\delta }}_{t}I\right)$$

### 3.5. Loss Function

Similar to most diffusion models, our model is trained by optimizing the evidence lower bound (ELBO), which can be formulated as:

(11) $$\begin{array}{l} ELBO=-E_{q}\left(D_{KL}\left(q_{BB}\left(x_{T}|x_{0},y\right)||p\left(x_{T}|y\right)\right)\right)\\ +\sum_{t=2}^{T}D_{KL}\left(q_{BB}\left(x_{t-1}|x_{t},x_{0},y\right)||p_{\theta }\left(x_{t-1}|x_{t},y\right)\right)\\ -\log p_{\theta }\left(x_{0}|x_{1},y\right) \end{array}$$

First, we only considered the first conditional control introduced by the Brownian bridge strategy. Since we fix the endpoint of the forward diffusion process, making $x_{T}=y$, the first term in Equation (11) can be treated as a constant and thus ignored. By utilizing Equations (4) and (8), along with Bayes’ theorem and the properties of Markov chains, we can express the posterior probability of the second term in Equation (11) as follows:

(12) $$\begin{array}{l} q_{BB}\left(x_{t-1}|x_{t},x_{0},y\right)=\frac{q_{BB}\left(x_{t}|x_{t-1},y\right)q_{BB}\left(x_{t-1}|x_{0},y\right)}{q_{BB}\left(x_{t}|x_{0},y\right)}\\ =N\left(x_{t-1};\tilde{\mu_{t}}\left(x_{t},x_{0},y\right),\tilde{\delta_{t}I}\right) \end{array}$$

According to [36,44], we can express $\tilde{\mu_{t}}\left(x_{t},x_{0},y\right)$ by reparameterization and simplification as follows:

(13) $$\mu_{\theta }\left(x_{t},y,t\right)=c_{xt}x_{t}+c_{yt}y+c_{\epsilon t}\epsilon_{\theta }\left(x_{t},t\right)$$

where $\epsilon_{\theta }$ is the noise distribution to be predicted by the neural network, and the specific forms of $c_{xt}$, $c_{yt}$, and $c_{\epsilon t}$ are as follows:

(14) $$\begin{array}{l} c_{xt}=\frac{\delta_{t-1}}{\delta_{t}}\frac{1-m_{t}}{1-m_{t-1}}+\frac{\delta_{t|t-1}}{\delta_{t}}\left(1-m_{t-1}\right)\\ c_{yt}=m_{t-1}-m_{t}\frac{1-m_{t}}{1-m_{t-1}}\frac{\delta_{t-1}}{\delta_{t}}\\ c_{\epsilon t}=\left(1-m_{t-1}\right)\frac{\delta_{t|t-1}}{\delta_{t}} \end{array}$$

Finally, by combining the above equations, Equation (11) can be simplified to:

(15) $$E_{x_{0},y,\epsilon }\left[c_{\epsilon t}||m_{t}\left(y-x_{0}\right)+\sqrt{\delta_{t}}\epsilon -\epsilon_{\theta }\left(x_{t},t\right)|{|}^{2}\right]$$

where $\sqrt{\delta_{t}}\epsilon$ is random noise to enhance the robustness of the neural network model. Then, using $\overset{\wedge }{y}$ to denote the encoded image after interpolation, we can obtain the final loss function of DCLTV as follows:

(16) $$E_{x_{0},y,\epsilon }\left[c_{\epsilon t}||m_{t}\left(y-x_{0}\right)+\sqrt{\delta_{t}}\epsilon -\epsilon_{\theta }\left(x_{t},\overset{\wedge }{y},t\right)|{|}^{2}\right]$$

### 3.6. Self-Attention Mechanism

Since the Transformer model [29] was proposed, it has gained prominence in large language models and image processing, becoming one of the most popular and effective models in the field of NLP and computer vision. The self-attention mechanism is the core of Transformer, which reduces the dependence of the original attention mechanism on external information and realizes a modeling approach that effectively captures global context information through a triplet $\left(key,query,value\right)$. The self-attention mechanism can better capture the internal correlation of data or features. It computes attention scores on the input itself using the scaled dot-product attention calculation as follows:

(17) $$\mathrm{Attention}\left(Q,K,V\right)=\mathrm{softmax}\left(\frac{QK^{T}}{\sqrt{d_{k}}}\right)V$$

thereby identifying the importance and relationships of different parts. To enhance our model to better recognize the background and various objects in the image for more accurate restoration, we incorporated the self-attention mechanism into our neural network.

**Figure 5:**
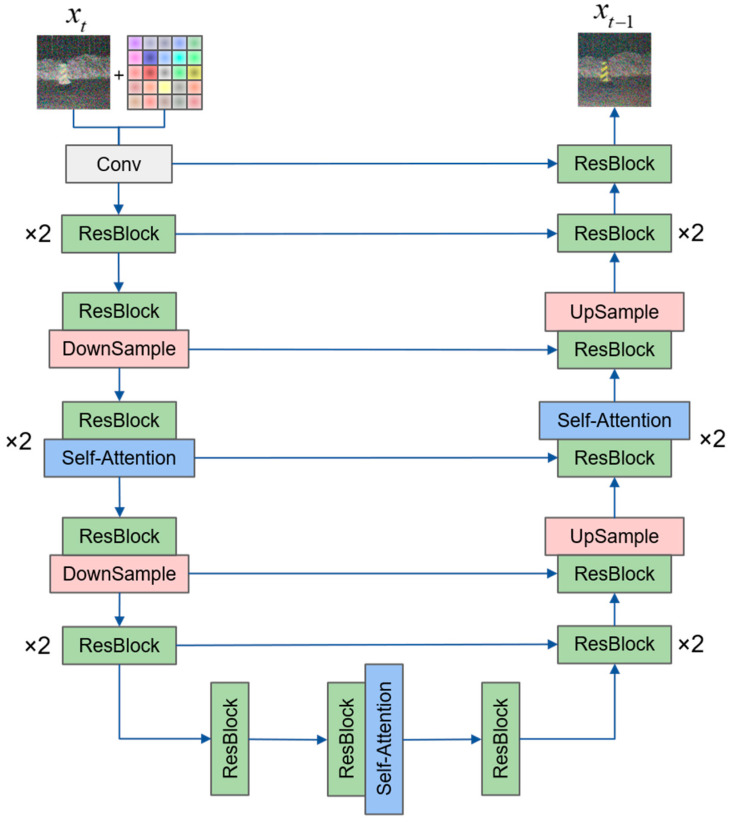
The neural network structure of DCLTV.

Figure 5 illustrates the structure of the neural network adopted by DCLTV. The self-attention mechanism has been applied to the intermediate layers within the downsampling process positioned on the left side of the network, the upsampling process located on the right side, and the connection part situated at the bottom, aiming to enhance the ability of the model to recognize and restore edge and color features in high-dimensional space.

## 4. Experiments and Results

In this section, we briefly describe our experimental setup, experimental environment, evaluation metrics, and baseline models. We also conducted ablation experiments to verify the effectiveness of the improvements and analyzed the results both quantitatively and qualitatively.

### 4.1. Experimental Setup

#### 4.1.1. Experimental Environment and Hyperparameters

We employed the Adam [51] optimizer to train the DCLTV model with a batch size of 4 and a fixed learning rate of 1 × 10^−4^. The training and validation processes were conducted using the laser-visible image dataset constructed in Section 2. Table 1 shows the other settings and environmental parameters.

During the sampling phase of DCLTV, we integrated the DDIM method [52] to expedite the inference process. By employing a non-Markovian approach, we effectively reduced the original inference steps from T=1000 to T=200, which not only ensures the rapidity of inference, but also maintains the quality of the generated outputs.

#### 4.1.2. Evaluation Metrics

We select two prevalently utilized metrics in image generation, Fréchet Inception Distance (FID) and Learned Perceptual Image Patch Similarity (LPIPS), to evaluate the quality, clarity, and fidelity of the generated images.

**FID:** This metric assesses the performance of generative models by comparing the distribution differences between generated and real images in feature space. Through a pre-trained Inception network, it extracts features separately from a set of real images and a set of generated images, obtaining their representations in feature space, and then calculates the mean and covariance matrix of these representations and uses the Fréchet distance formula to measure the difference between these two sets of means and covariance matrices. Fréchet distance [53] is a measure of similarity between two probability distributions, with a smaller FID value indicating that the generated images are closer to the real images in distribution, thus exhibiting higher quality.

**LPIPS:** This metric evaluates image quality and similarity by employing deep learning methods to simulate human sensitivity to image differences. The core idea of LPIPS is to use a pretrained convolutional neural network (CNN) to extract features from input images (generated and target images), obtaining feature representations at different levels. It compares feature representations of the two sets of images and calculates the difference, typically involving weighted and normalized feature maps, followed by computation of the Euclidean distance. The weighted sum of the differences at various levels is taken as the LPIPS value, which measures the similarity between generated and target images and accurately captures the differences perceived by the human visual system. A smaller LPIPS value indicates a smaller visual difference between the generated and target images.

To obtain more accurate and reliable FID and LPIPS values, we generated five samples for each laser image input in the test set for calculating the average error, thereby enabling more precise evaluation of the models.

#### 4.1.3. Baseline Models

We selected several representative baseline models from the field of cross-modal translation for visible images to compare with DCLTV, including Pix2pix [11], BigColor [13], CT2 [15], ColorFormer [14], and DDColor [16]. Pix2pix and BigColor are GAN-based models, while CT2, ColorFormer, and DDColor are Transformer-based models.

### 4.2. Comparative Results

#### 4.2.1. Qualitative Comparison

**Figure 6:**
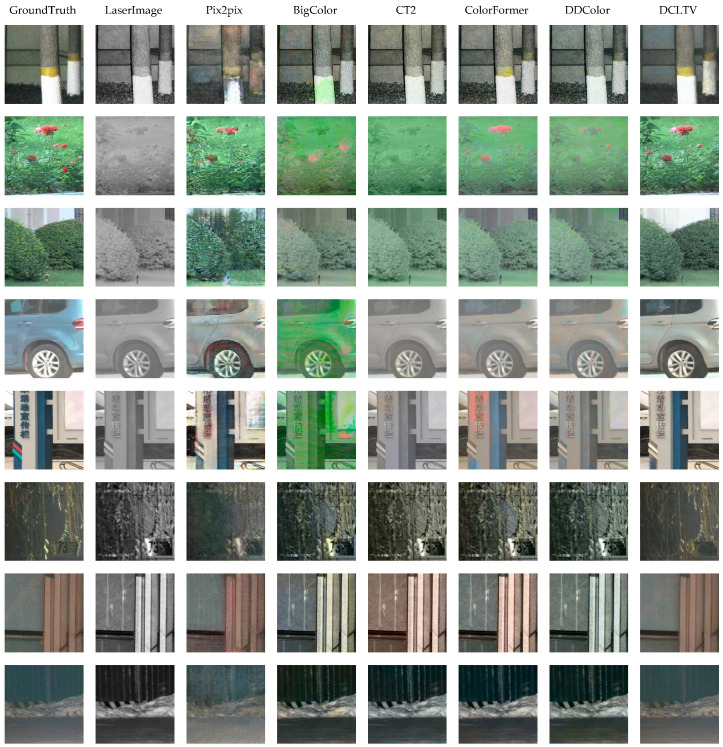
Qualitative results for baseline models and DCLTV.

Figure 6 presents the qualitative results of our DCLTV compared to the baseline models. It is evident that the generated images of Pix2pix are generally consistent with the real images, but exhibit blurred edge features and low fidelity. BigColor struggles to learn mapping from the laser image domain to the visible image domain. CT2 manages to recover the main colors of some scenes, such as the flowers in the second and third rows and the scattered branches in the sixth row. ColorFormer shows relatively satisfactory translation results, but still leans towards the edge features of laser images, indicating low fidelity. The performance of DDColor is similar to that of CT2, excelling in certain details, such as identifying a hint of red flower features in the second row and restoring some yellow stripes on the warning sign in the last row. In contrast, our DCLTV demonstrates more accurate translation results for most scenes and targets. In natural environments, DCLTV can accurately distinguish plants from backgrounds and does not suffer from color bleeding issues like other models. When restoring building images, DCLTV has the ability to identify and eliminate the non-natural reflections caused by laser illumination. Meanwhile, the images restored by DCLTV are more akin to visible images compared to other models in terms of human visual perception. This improved performance can be attributed to the dual-condition constraints, the self-attention mechanism, and the diverse generation of the diffusion model.

#### 4.2.2. Quantitative Comparison

In the quantitative comparison, we calculated the FID and LPIPS values for the selected baseline models and DCLTV. Table 2 presents the quantitative results for each model. As shown in Table 2, compared to Pix2pix, BigColor, CT2, ColorFormer, and DDColor, the FID value of DCLTV decreased by 129.072, 55.414, 61.599, 29.229, and 62.585, respectively, while the LPIPS value decreased by 0.107, 0.156, 0.127, 0.170, and 0.141, respectively. Overall, our model achieves the best performance in terms of both FID and LPIPS scores, demonstrating a significant advantage over the other models.

### 4.3. Ablation Study

In this section, we describe an ablation study of the dual condition and the self-attention mechanism within the model to validate the effectiveness of each improvement.

We conducted experiments in three scenarios: single-condition, dual-condition, and dual-condition with a self-attention mechanism. The quantitative experimental results are presented in Table 3.

The quantitative results show that, when employing the Brownian bridge strategy for the first conditional control, the model benefits from integrating the condition into the diffusion process, allowing it to directly learn mapping between the laser image domain and the visible image domain. The results for DCLTV(1C) already outperform the other baseline models shown in Table 2, with a minimum FID reduction of 2.17% and a minimum LPIPS reduction of 12.35%. Adding the interpolation features of laser images as the second condition enhanced the ability of the model to learn the color and edge features of scenes and targets, further reducing both metrics. Compared to DCLTV(1C), the FID value of DCLTV(2C) decreased by 8.47% and the LPIPS value by 10.56%. The final addition of the self-attention mechanism enabled the model to better capture the intrinsic relationships between scenes and targets, enhancing its capabilities for color and edge recovery. DCLTV(2CwithSAttn) achieved the best performance among the three scenarios, with an FID value of 154.74 and an LPIPS value of 0.379, representing improvements of 6.11% and 0.53% over DCLTV(2C), respectively.

**Figure 7:**
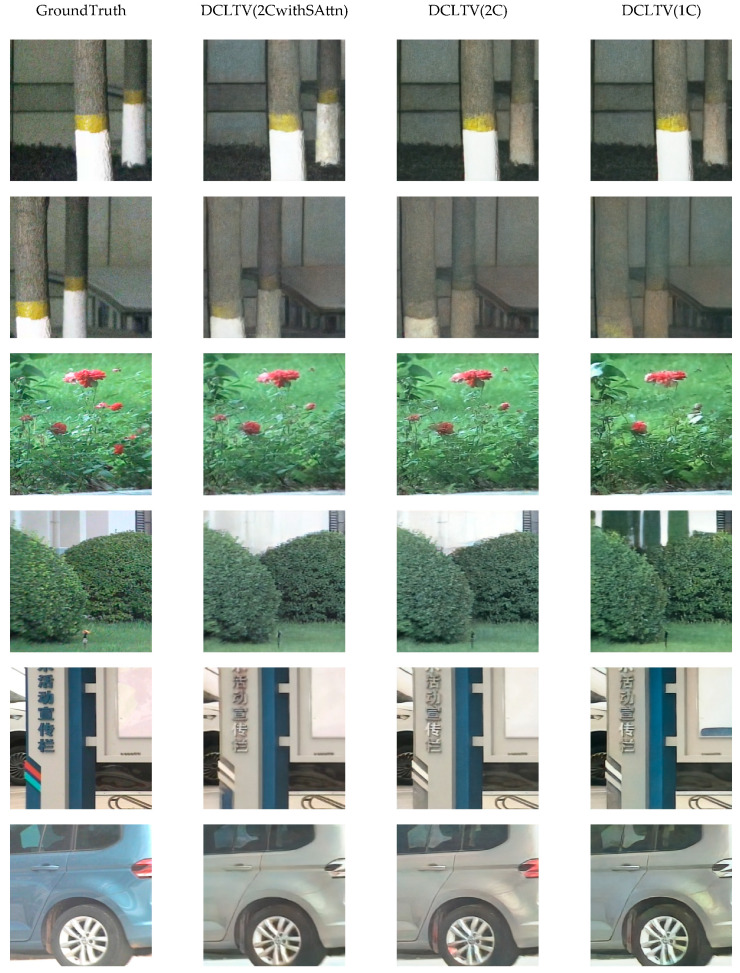
The images generated in the ablation study.

The images generated by the three scenarios are shown in Figure 7. After adding the first conditional control, the generated images were more visually consistent with real images compared to those images produced by the baseline models described in Section 4.2.1. However, the images generated by DCLTV(1C) still exhibit certain issues, such as color deficiency, color bleeding, and blurred edge features. With the addition of the second conditional control and the self-attention mechanism, these problems were somewhat mitigated, and the details of the scenes and targets were improved. By leveraging the global modeling and high-level feature extraction capabilities of the self-attention mechanism, DCLTV(2CwithAttn) further mitigated the problem of color bleeding and achieved the best translation performance. Subjectively, the effectiveness of the improvements obtained using DCLTV was further validated.

## 5. Conclusions

In this study, we explored the field of laser-visible image translation and proposed the DCLTV model, which introduces a diffusion model to achieve this novel task. In order to generate images that align more closely with the specific requirements of tasks, we incorporated dual-condition constraints into the traditional diffusion model for more deterministic generation. Inspired by the Brownian bridge, we integrated the conditional image directly into the diffusion process as the first condition, enabling the diffusion model to directly learn the mapping from the laser image domain to the visible image domain. To further restrict the generative randomness of the diffusion models, we proposed an interpolation-based conditional injection method as the second condition to enhance the capability of the model to recognize and restore the color and edge features of scenes and targets within the images. Compared to five representative baseline models, both subjectively and objectively, DCLTV achieves the best performance. The quantitative results showed that DCLTV achieved reductions of at least 15.89% in FID value and at least 22.02% in LPIPS value.

## Figures and Tables

**Table 1:** Environmental settings and parameters.

Hardware and Software	Parameters
Operating System	Ubuntu 22.04
CPU	Inter(R) Xeon(R) Gold 5218R
GPU	NVIDIA RTX A6000
Video Memory	48 GB
Deep Learning Framework	Pytorch 2.1.0

**Table 2:** Quantitative results for baseline models and DCLTV.(The downward arrow signifies that a lower value for this metric is preferable; highest score marked in bold font).

Model	FID ↓	LPIPS ↓
Pix2pix	283.812	0.486
BigColor	210.154	0.535
CT2	216.339	0.506
ColorFormer	183.969	0.549
DDColor	217.325	0.520
DCLTV	**154.740**	**0.379**

**Table 3:** Quantitative results from the ablation study. (The downward arrow signifies that a lower value for this metric is preferable; highest score marked in bold font).

Model	FID ↓	LPIPS ↓
DCLTV(1C)	180.068	0.426
DCLTV(2C)	164.809	0.381
DCLTV(2CwithSAttn)	**154.740**	**0.379**

## Data Availability

The data that support this study are proprietary and may only be provided with restrictions.
